# The accuracy of preliminary diagnoses made by paramedics – a cross-sectional comparative study

**DOI:** 10.1186/s13049-020-00761-6

**Published:** 2020-07-23

**Authors:** Outi Koivulahti, Miretta Tommila, Elina Haavisto

**Affiliations:** 1 Department of Nursing Science, University of Turku, Department of Nursing Science 20014 University of Turku, Turku Finland and Satakunta Central Hospital, Sairaalantie 3, 28500 Pori, Finland; 2 Department of Perioperative Services, Intensive Care Medicine and Pain Management, University of Turku and Turku University Hospital, PO Box 52, 20521 Turku, Finland

**Keywords:** Clinical decision making, Emergency medicine, Paramedic, Pre-hospital nurse, Preliminary diagnosis

## Abstract

**Background:**

Clinical decision-making skills of paramedics have been emphasized because of the growing complexity of emergency medicine nursing. A preliminary diagnosis made by a paramedic has an essential role in directing the subsequent care. An accurate preliminary diagnosis improves the patient’s outcome. The research in this area is relatively scarce and there are no previous studies in Finland describing the accuracy of preliminary diagnoses made by paramedics. The aim of this study was to evaluate whether paramedics are making accurate preliminary diagnoses for the patients they are transporting to hospital. In addition, the aim was to describe the variables related to an accurate preliminary diagnosis.

**Methods:**

A cross-sectional comparative approach was used and conducted through a questionnaire to gather data from the paramedics. A total of 71 paramedics participated in the study and 378 patient cases were included. The paramedics were asked to describe the basic information of a case, to state their preliminary diagnosis, and give their own educational background. The accuracy of the paramedic’s preliminary diagnosis was compared with the discharge diagnosis of the ED physicians retrieved from hospital’s patient records. Logistic regression analysis and a binomial test were used to test the statistical significance.

**Results:**

The agreement between the paramedics’ preliminary diagnosis vs. hospital diagnosis was 70% (*n* = 261). Diagnostic accuracy varied according to the medical condition from mental diseases and intoxication (*86%, p = 0,000*), cerebral strokes *(81%, p = 0,007)* to infections (*31% p = 0,029*). The educational background of a bachelor-degree-level paramedic (*p = 0,016, 95% Cl 1,7-139,6*) and a good self-assessment value (p = 0,*003, 95% Cl 1,2-2,7*) were related to making a correct diagnosis.

**Conclusions:**

Paramedics are able to determine preliminary diagnoses at satisfactory level. The relationship between educational background and diagnostic accuracy suggests that there is a definitive need for a specific pre-hospital nursing education.

## Background

The purpose of the Emergency medical services is to assess patients suffering from acute illnesses or injuries, provide the proper treatment, and transport patients to a definitive care if necessary [1]. In order to fulfill these expectations, paramedics must be able to estimate the condition of patients, and to already establish a preliminary diagnosis in order to initiate appropriate care in the field, or during transportation. Although an interim stage on the way towards an accurate diagnosis made by a physician, a preliminary diagnosis has an essential role in directing the care and judging the urgency of the transportation.

The work of paramedics has become more complex due to the increase in emergency calls [2, 3], demographic changes and new healthcare technologies that enable intensive pre-hospital treatments [4–6]. Because of these facts, emphasis has now been placed on the clinical decision-making skills of paramedics. Determining an accurate preliminary diagnosis has implications for the quality and safety of care and improving patients’ outcomes [7, 8]. Making a wrong preliminary diagnosis can lead to inadequate treatment or adverse events for patients [7, 9, 10].

Despite the crucial nature of this work performed by paramedics, there are only a few papers covering this area [10–14] and the results vary widely. Cummins and co-workers have investigated the accuracy of preliminary diagnoses made by paramedics with an overall accuracy of 70% [12]. Two studies evaluated paramedic diagnoses in respiratory emergencies and the authors found that when paramedics determined the underlying etiology in breathing difficulties, either cardiac, respiratory or other disease, they had an agreement of 81,1% [11] and when making preliminary diagnoses they had an accuracy of 64% [10]. In an intervention study, paramedics identified sepsis using a screening tool with an accuracy of 78,2% [13] and an ST-segment elevation myocardial infarction was detected with reasonable accuracy [14, 15]. Studies show that paramedics with a longer education are better at clinical decision-making [6, 10, 15]. However, there are no international standards or regulations for paramedics or prehospital emergency care nurses [16] which makes it difficult to generalize the results of the studies.

The aim of this study was to evaluate the overall accuracy of the paramedics’ preliminary diagnosis and the accuracy is divided into categories by disease or injury. In addition, the aim was to describe the related variables to an accurate preliminary diagnosis.

## Methods

### Study design

A cross-sectional comparative study design was conducted using a questionnaire to gather data from the paramedics. Hospital records were used to collect the physician’s diagnoses.

### Setting and sample

The participants of this study were all paramedics working in different levels of emergency practice. In Finland, where this study was conducted, the emergency medical service has three-tiers consisting of first response units, basic and advanced life support level ambulances and physician staffed units [17]. Paramedics assess their patients independently and evaluate their need for care. Physician units only participate the highest priority calls, however, paramedics have the opportunity to consult a physician if they need medical support. Prehospital electrocardiograms are systematically electronically transmitted to hospital for a later interpretation of a physician.

In Finland, the professional qualifications for ambulance crew members at the basic level are: at least one paramedic must be registered health professional with prehospital emergency care training and the other must be a health professional or a fireman. At the advanced level one paramedic must be a registered nurse with additional prehospital emergency care training or a specific registered prehospital emergency care nurse (bachelor-level degree) and the other must be a health professional or a fireman [18].

This study was conducted in one hospital district in Finland. The hospital district covers an area of 7820 km2 and has approximately 220,000 inhabitants. The local Emergency Medical Service consists of 17 advanced level ambulances staffed with a total of 158 paramedics. Annually there are over 29,000 emergency calls leading to an ambulance response. According to hospital records, an estimated 1000–1200 patients are transported by EMS to the central hospital monthly. Approximately 85% of the patients are transported without a referral but the exact numbers are not known due to the fact that they are not systematically documented by the central hospital.

### Data and data collection

In this study we used two datasets. The first dataset was a semi-structured questionnaire developed for this specific study. The questionnaire was based on a systematic literature review conducted for this study and on the national targeted learning outcomes of paramedic education. There was an expert panel with two emergency medical doctors, three paramedics and a healthcare teacher who commented on the questionnaire in two rounds and then reached a consensus by adding one more question and modifying two others. The expert panel also recommended the use of an ICPC-2 classification to state the preliminary diagnosis so that the answers would be less ambiguous. The questionnaire consisted of eleven questions in two parts (Table 1). The preliminary diagnosis was stated according to the ICPC-2 classification or with the paramedics own words if not found among the ICPC-2 classification codes.

**Table 1:** The questionnaire

First part (information of the emergency call)	Second part (personal information)
**Date and time**	**Level of practice (basic/advanced)**
**Priority code of the convoy (A/B/C/D)**	**Work experience (years and months)**
**Patient’s social secure number**	**Educational background (multiple choice)** **־ degree level** **־ continuing education in acute nursing**
**Physician consultancy (yes/no)**	**Self-assessment value given for the preliminary diagnosis (4-10)**
**Preliminary diagnosis (ICPC-2 code / own words)**	

Paramedics were asked to complete a questionnaire concerning patients transported to the emergency department of the central hospital between 30.3.2018 and 30.4.2018. Emergency calls and patients without a referral were included. A total of 415 questionnaires were returned of which 378 were included in the study. Questionnaires with an invalid social security number as a patient identification (*n* = 15) or a missing preliminary diagnosis (*n* = 1) were excluded. In addition, one questionnaire completed outside the data collection time (*n* = 1) was excluded.

The second dataset was formed from the hospital’s electronic patient records in which the physician’s diagnosis, according to ICD-10 classification, was collected at the end of the caring period in the emergency department. Furthermore, only the diagnosis concerning this specific hospitalization was included. In this phase, patient cases with a missing physician’s diagnosis (*n* = 17) or patient cases with a referral (*n* = 2) were excluded.

Patient identification details from the questionnaires were extracted to a database (Microsoft Excel©). The physician’s diagnosis was audited manually from the hospital’s patient records by the patient’s identification number and a date. To avoid bias the physician’s diagnosis was first extracted to a spreadsheet and the preliminary diagnoses from the questionnaires were extracted later and an interpretation of the accuracy was then made. After comparing diagnoses the data was de-identified. All diagnoses were divided into categories and subcategories which described the pathophysiology of the disease or injury.

### Statistical analysis

The data was analyzed with IBM SPSS Statistics® (version 25). Descriptive statistics were described with frequencies and percentages. The accuracy of diagnoses in certain categories was analyzed by using a two-tailed binomial test in order to ascertain whether the paramedics were able to make preliminary diagnosis more accurately depending on the type of disease. Logistic regression analysis was used to test the statistical significance of the relationship between background variables and the accuracy of the preliminary diagnosis. The level of statistical significance was considered to be a *p* value of less than *0.05*.

### Ethical considerations

Ethical approval for this study was received from the Ethics Committee of Satakunta Hospital District (02.03.2018). Additionally, the study permission for the data gathering from the paramedics and from the hospital records was obtained from the central hospital (statement codes: ETMK 33/2018, 37/2018). For the paramedics, their participation in the study was completely on a volunteer basis with an assurance of anonymity. The data collection did not have any impact on the care of patients and informed consent of the patients was not required because of the de-identification of the data.

## Results

### Characteristics of the participants

A total of 71 (45%, *n* = 158) paramedics participated in the study. Their level of practice was basic life support (4.8%) and advanced life support (95.2%). The median number of the work experience in years was 7.0 (IQR 7.1). The educational background of the paramedics varied (Table 2) and two paramedics did not complete the background section of the questionnaire. The number of questionnaires returned was 378; this equals the number of patient cases included in the study.

**Table 2:** Educational background of the paramedics

Educational background (degree)	Paramedics (n)	Paramedics (%)	Patient cases (n)
**Bachelor degree in nursing**	48	68	241
**Bachelor degree in pre- hospital nursing**	15	21	111
**Practical nurse (upper secondary level)**	6	8	17
**No education details**	2	3	9

### Characteristics of the patient cases

Most of the ambulance calls (87%) were coded with low priority and the remainder (13%) were high priority calls. The paramedics consulted a physician in 31% of the patient cases. Preliminary diagnoses concerning cardiovascular diseases (16%) and respiratory diseases (14%) were the most common in this study (Table 3).

**Table 3:** The accuracy of preliminary diagnoses by categories

Cases by categories (n)	Accuracy of the preliminary diagnoses (%)	Statistical signifigance (***p***-value)*
**Cardiovascular emergencies (65)**	71	*0.001*
**Respiratory emergencies (58)**	57	0.358
**Musculoskeletal injuries (37)**	76	*0.003*
**Infectious diseases (36)**	31	*0.029*
**Mental health and drug related (35)**	86	*0.000*
**Gastrointestinal and endocrinological emergencies (29)**	69	0.061
**Nervous system (29)**	79	*0.002*
**Soft-tissue injuries (27)**	85	*0.000*
**Cerebral strokes (21)**	81	*0.007*
**General deterioration (18)**	67	0.238
**Traumatic brain injuries (13)**	77	0.092
**Nasal or gastrointestinal bleeding (non acute) (10)**	100	*0.002*

* Binomial Test (2-tailed) Test Proportion 0.5

### The accuracy of preliminary diagnoses by patient case type

The correct preliminary diagnosis was determined in 263 of the patient cases resulting in an overall accuracy of 70%. The accuracy of preliminary diagnosis was strongly related to the patient’s case type (Table 3). Paramedics determined the preliminary diagnoses with the highest accuracy in cases related to mental health and drugs, soft-tissue injuries and cerebral strokes. Among the most common type of emergencies were situations related to cardiovascular diseases, which the paramedics determined with an accuracy of 71%, though, cardiac arrhythmias were recognized with an accuracy of 93%. However, the level of accuracy was most deficient in respiratory emergencies and infectious diseases (Table 3).

In the infectious diseases category, the paramedics recognized influenzas and urinary infections with an accuracy of 13 and 20%, respectively. The typical preliminary diagnosis they determined in these cases was either fever or general weakness. In respiratory emergencies, the most common diagnosis made by the physician was pneumonia, which the paramedics recognized with an accuracy of 54%.

### Background variables related to an accurate preliminary diagnosis

The educational background of the paramedic and the given self-assessment value of the preliminary diagnosis were statistically significant to the accuracy of the preliminary diagnosis (Table 4). Both a bachelor level degree in pre-hospital nursing and continuing education in advanced level pre-hospital care increased the accuracy of the preliminary diagnosis. If a paramedic gave a good self-assessment value to the preliminary diagnosis, this determined that the odds would be higher for the preliminary diagnosis to be accurate (Table 4).

**Table 4:** Background variables

Background variables	Cl. 95% (lower-upper)	OR (Odds Ratio)	Statistical significance (***p***-value)*
**Degree programme**			
**Bachelor degree in nursing**	0.374–14.310	2.315	0.367
**Bachelor degree in pre-hospital nursing**	1.652–139.572	15.184	*0.016*
**Upper secondary level degree in practical nursing**	0.425–44.261	4.339	0.215
**Continuing education**			
**Advanced level pre-hospital care (30 ECTS)**	1.609–15.173	4.941	*0.005*
**Leadership in EMS (30 ECTS)**	0.115–0.739	0.292	*0.009*
**Other study in acute nursing**	0.963–8.170	2.805	0.059
**Given self-assessment value**	1.232–2.728	1.833	*0.003*
**Work experience in pre-hospital care**	0.759–1.482	1.060	0.733
**Priority of the conveyance**	0.750–3.249	1.561	0.234

* Logistic regression analysis

## Discussion

The increasing number of emergency calls and especially non-urgent calls [3, 5] have made it challenging yet crucial for paramedics to assess the care needs of patient and make the proper choices according to the patient’s condition. If the pathophysiology of the disease remains unclear or the emergency situation demands a fast evacuation and transportation, the patient must be treated based on their symptoms and relieving them [6–8]. However, the significance of an accurate preliminary diagnosis is emphasized when long distances are involved and in areas of dispersed settlement when it becomes essential for ensuring effective treatment and definitive care.

### The accuracy of preliminary diagnoses

The aim of this study was to evaluate the accuracy of the preliminary diagnoses made by paramedics and the variables related to the diagnosis. The overall accuracy of the preliminary diagnoses made by the paramedics was 70%, which has also been reported in previous studies [12]. This can be considered satisfactory allowing for the limited information, lack of diagnostics in the field and the challenging environment, even though the majority of the cases in our study were non urgent. Despite the lack of the time pressure, the paramedics are challenged by a large variety of complex cases including aged patients with multiple comorbidities and general weakness.

The highest level of accuracy, that of mental health and drug related emergencies and soft-tissue injuries, seems obvious due to the nature of these situations. Nevertheless, in one other study [12] the levels of accuracy (58, 75% respectively) in these categories were remarkably different. The high level of recognizing cerebral strokes (81%) is extremely important because of the survival and recovery of the brain and functioning of the patient [19] and the result is in agreement with other studies [12], although, in this present study, the categories were more specific including only ischemic and hemorrhagic strokes and transient ischemic attacks. The overall accuracy of the preliminary diagnoses in cardiovascular diseases was satisfactory, however it must be emphasized that the recognition of cardiac arrhythmias was on a very high level, suggesting that the paramedics can consistently interpret electrocardiogram in this area. The effect of prehospital delays in survival in acute illnesses e.g. ST-segment elevation myocardial infarction [14, 20] patients is unquestionable, which makes it crucial for paramedics to interpret electrocardiograms.

The level of accuracy was lowest in respiratory emergencies (57%) and infectious diseases (31%). The result in respiratory emergencies is even lower than reported in other studies in which the level was 65% [10, 12]. The unsatisfactory level of accuracy in preliminary diagnoses may be a consequence of the complicated pathophysiology of respiratory diseases [10–12] or suggest an inadequate examining of the patient. Identifying respiratory diseases and understanding the anatomy and pathophysiology of respiratory system is an essential part of paramedic competencies due to the fact that emergencies related to respiratory diseases or shortness of breath are one of the most common emergency calls [10, 11].

The recognition of infectious diseases seemed lower compared to other preliminary diagnosis areas according to our results. The preliminary diagnosis of influenza proved to be especially difficult but that could partly be explained by the fact that there was no code for influenza in the ICPC-2 classification list, although the participants were instructed to state the preliminary diagnosis with their own words if not found in ICPC-2 classifications. Moreover, urinary infections were not recognized either on a satisfactory level. Although common in the geriatric population, the symptoms of urinary infections may remain very unclear, and it can even be argued, that not all diagnoses of urinary infection made in the ED are necessarily accurate.

The most commonly used preliminary diagnoses that were used instead of a specific infection code were fever and general weakness, which could be considered reasonable options in this context. Ultimately, the most important aspect concerning infections is undoubtedly the early recognition of severe infections, especially sepsis, because early recognition improves the patient’s outcome [13, 21]. In these situations an accurate preliminary diagnosis must be determined. However, in our study, there were only a few cases of severe infections and no conclusions can be made concerning them. In addition, a more precise preliminary diagnosis might be needed, for example, when considering transportation decisions or the patients need to be isolated. For more accurate diagnostics, adding the possibility of point of care testing for paramedics [4] might be one solution.

### Education matters

The relationship between the bachelor degree in pre-hospital emergency nursing and the accuracy of the preliminary diagnosis was clear. The relationship between specific emergency nursing education and paramedic competencies in general has been reported in previous studies [6, 10, 15, 22]. In Finland, the bachelor degree in pre-hospital emergency nursing takes 4 years and concentrates on pre-hospital emergencies, however, the students are also registered as nurses. A bachelor’s degree in nursing does not give readiness for working in a pre-hospital setting but, as reported in the study results, the specialization courses in pre-hospital nursing increases the capability of a paramedic’s clinical decision making and the accuracy of their preliminary diagnoses.

The paramedics were asked to provide a self-assessment value to the preliminary diagnosis they determined. Overall, the paramedics were confident with the preliminary diagnoses they made. The results suggest that in cases of a low self-assessment value the actual physician’s diagnosis was also likely to be undefined or related to a general weakening without a specific diagnosis. Furthermore, the paramedics with a work experience of less than 5 years were less confident about their preliminary diagnoses than their more experienced colleagues, nevertheless, the amount of work experience was not statistically significant as regards the accuracy of preliminary diagnoses. The higher self-assessment values may be explained by professional growth and increased self-confidence and it is important to give enough support to novice paramedics in order to reinforce their clinical decision-making.

### Limitations

The main limitation of this study and generalization of the results is the sample size and the possibility of bias. The number of patient cases included in this study was 378 and all the cases that meet the inclusion criteria were recruited. The size of the sample was sufficient for the patient cases to be divided in statistically representative categories by the type of the emergency or illness and the cases also represent the national distribution of EMS’s transportation codes. We were not, however, able to make prior power calculations since there were only estimations available for the numbers of patients transported by ambulances to the emergency department. However, based on the estimations we achieved a response rate of approximately 40%.

The data was collected in the area of one hospital district during 1 month in the spring, and this also reduces the generalization of the results as more of a certain type of illnesses e.g. influenzas may occur at this time of year. Furthermore, the sample covers well the variety of different cases, except pediatrics or incidents of labor which were not included in this study due to the fact that they are not treated in the emergency department.

Second, the questionnaire used in this study was self-developed since there was no suitable questionnaires available. The questionnaire was not pilot-tested but there was an expert panel and two rounds of assessment to ensure the validity and clarity of the instrument. The questionnaire was designed to be semi-structured to maintain the homogeneity of the answers.

The third limitation of this study is the use of the diagnosis made by physician’s as a reference standard to determine the accuracy of the paramedic’s preliminary diagnosis. A physician’s diagnosis may not always be completely accurate and there is a possibility that the time between the preliminary and actual diagnosis and treatment given in the prehospital care may affect the patient’s condition and symptoms, thus resulting in a different diagnosis. We wanted to use the diagnosis at the patient’s discharge from ED and not the admission diagnosis due to the fact that the discharge diagnosis differs from the admittance diagnosis in every ninth patient [23]. Furthermore, the preliminary diagnoses were made by using the ICPC-2 classification, whereas the ED discharge diagnoses were made by using the ICD-10 classification. This may have affected the agreement analysis of the preliminary diagnosis and physician’s diagnosis since the ICPC-2 classification codes included more general descriptions of symptoms.

## Conclusions

Paramedics are able to determine preliminary diagnoses at satisfactory level. Furthermore, it must be emphasized that the level of accuracy varies by the type of illness or emergency. It seems that paramedics are able to determine preliminary diagnoses more accurately in cases with explicit findings; e.g. visible injuries, neurological symptoms or electrocardiogram abnormalities.

Less acute patients, which are usually the patients with unclear symptoms, more often remain without an accurate preliminary diagnosis. This should be observed since the number of low priority calls are increasing and the population is aging. The dilemma is how to educate paramedics in order to maintain a sufficient level of competence in acute emergencies, but also increase training in general weaknesses.

Furthermore, it is important to concentrate on the education of paramedics. Based on the results of this study, the paramedics with a bachelor-degree-education in emergency care nursing are achieving better preliminary diagnoses than paramedics with bachelor-degree in nursing. The nature of pre-hospital emergency care nursing is different from nursing in a hospital because of the need for a more independent medical interview, examination and assessment of the patient. Paramedics need more knowledge in anatomy and pathophysiology in order to clinical hypotheses and make clinical decisions.

## Data Availability

The datasets generated and analysed during this study are not publicly available due to the restrictions in the study approval.

## Abbreviations

ED: Emergency department
EMS: Emergency Medical Service
ICD-10: International Classification of Diseases, 10th edition
ICPC-2: International Classification in Primary Care, 2nd edition
